# Case Report: Successful Use of Extracorporeal Therapies After ECMO Resuscitation in a Pediatric Kidney Transplant Recipient

**DOI:** 10.3389/fped.2020.593123

**Published:** 2020-12-15

**Authors:** Andrey Rybalko, Anna Pytal, Mikhail Kaabak, Nadejda Rappoport, Anuar Bidzhiev, Vasilii Lastovka

**Affiliations:** ^1^Intensive Care Unit, National Medical Research Center for Children's Health, Moscow, Russia; ^2^Organ Transplantation Department, National Medical Research Center for Children's Health, Moscow, Russia

**Keywords:** kidney transplantation, cardiac arrest, ECMO, immunosuppression, plasma exchange, CytoSorb, lipopolysaccharide adsorption

## Abstract

The combination of extracorporeal membrane oxygenation (ECMO) and extracorporeal blood purification in children is rarely used due to small total blood volumes, risks of hemodynamic instability and a negative association between volume of blood transfusion and patient outcome. To our knowledge, this is the first description of a multimodal extracorporeal detoxication in the setting of ECMO in a post-kidney-transplant child on immunosuppression. We describe a case of a 30-months old child, who was extracorporeally resuscitated after cardiac arrest during kidney transplantation surgery and additionally treated with a number of extracorporeal blood purification methods (plasma exchange, CytoSorb, and lipopolysaccharide adsorption) in the setting of immunosuppression therapy. This case report shows the successful use of multimodal extracorporeal therapies for a good patient outcome. The lack of response to CytoSorb therapy might suggest an occult infection and not necessarily failure of treatment.

## Introduction

Extracorporeal membrane oxygenation (ECMO) in refractory cardiac arrest secondary to arrhythmias in children increases patient survival to 42% compared to standard cardiopulmonary resuscitation (CPR) (1). ECMO stabilizes hemodynamics, provides controlled oxygen delivery to tissues, and prevents further post-CPR cardiac arrest (2). Extracorporeal therapies in children allow for clinical stabilization in a short period of time (3). However, ECMO adds to the severity of ischemia-reperfusion injury (IRI) and can play a negative role in multiorgan failure (MOF) development (4); transfusion in children, during ECMO is associated with higher in-hospital mortality (5).

Here we describe a case of using ECMO in conjunction with multimodal extracorporeal therapy in a successful treatment of an otherwise hopeless patient.

## Case Description

A 30-month-old 6.4 kg 71 cm boy with autosomal recessive polycystic kidney disease was maintained on dialysis since the second month of his life. Significant developmental deterioration was observed during the previous 12 months of his life. The patient lost his walking ability, needed intermittent mechanical ventilation that led to a tracheostomy 6 months prior to transplantation. One week prior to transplantation anti-A isoagglutinin titer was 1:16, anti-B isoagglutinin titer was 1:2. Extracorporeal isoagglutinin removal was not performed. Immunosuppression induction was alemtuzumab and eculizumab; maintenance immunosuppression was tacrolimus and mycophenolate mofetil (MMF) in regimens previously described in publications (6, 7). We performed a bilateral nephrectomy and a right kidney transplant from his ABOi 63-year-old grandmother, AB to O.

Patient suffered a cardiac arrest due to hyperkalemia (12.9 mmol/l) during the advanced stage of surgery prior to graft reperfusion. As the standard CPR was ineffective, the child was started on ECMO with the right common carotid artery and jugular vein cannulation. Centrifugal pump (Deltastream DP3, oxygenator Hilite 2400 LT, Medos Medizintechnik, Germany), rotation of 4,500–6,000/min allowed blood flow of 1.5–2.5 l/min/m^2^. After 27 min of ECMO sinus rhythm recovered. Vascular anastomoses were completed, and graft reperfusion started while on ECMO. The abdomen was left open to reduce abdominal pressure and restore venous return. Upon ICU admission the ECMO flow was suboptimal (1.4 L/min/m^2^), inotrope support included high doses of dopamine (15 mcg/kg/min) and epinephrine (0.6 mcg/kg/min). The blood pressure was 95/78 with a mean arterial pressure of 83 mmHg, heart rate was 135/min. The child had a low cardiac index (1.35–1.5 L/min/m^2^) and severe coagulopathy (INR 2.52, aPTT 104.7 s, fibrinogen 0.6 g/L); there was bleeding from the cannulas exit sites and the surgical wound.

Despite severe IRI, we maintained adequate brain and kidney graft perfusion (assessed by near infrared spectroscopy, Table 1). Severe IRI resulted in elevated ALT, AST, LDH, and CK levels, blood counts revealed thrombocytopenia 29 × 10^9^/L and low hemoglobin 76 g/L. Defibrillation was required twice during the first 24 h post-op for ventricular tachycardia. It took us 15 h to achieve adequate ECMO flow and stable hemodynamics with fluid loading, vasopressor infusions, blood components transfusion, and other such methods.

**Table 1:** Daily clinical pattern of the patient.

**Parameter, Units**	**Day 0**	**Day 1**	**Day 2**	**Day 3**	**Day 4**	**Day 5**	**Day 6**	**Day 7**	**Day 8**	**Day 9**	**Day 10**	**Day 11**	**Day 12**
PE, circulating plasma volumes		2	1										
CytoSorb, hours			12	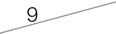									
Toraymyxin PMX-20R, hrs				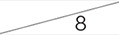	8						8		
HR, bpm	125	135	139	155	119	86	102	87	78	115	110	100	125
CVP, mmHg	35	24	14	14	14	15	14	15	14	14	12	10	13
Systolic BP, mmHg	84	95	73	84	64	92	77	112	91	116	108	125	124
Diastolic BP, mmHg	57	78	47	58	40	51	55	61	57	79	64	73	84
MAP, mmHg	69	83	58	70	49	65	63	83	64	97	83	86	85
Dopamine, mcg/kg/min		15	15	15	6	5	8	5	7	7	7	5	6
Norepinephrine, mcg/kg/min				0,8	0,4	0.05							
Epinephrine, mcg/kg/min		0.6	0.25	0.7	0.2	0	0.08	0.02		0.03	0.03	0.04	
Vasoactive inotrope support (VIS) index		75	40	165	66	10	16	7	7	7	10	9	
ECMO flow, ml/min	510	460	710	780	850	270	220						
ECMO, rotations per minute	5,900	5,150	4,550	4,800	4,600	3,550	3,450						
Flow index L/min/m^2^	1.5	1.35	2.08	2.29	2.5	0.8	0.64						
RBC, units (1 unit−100 ml)	2	1	1	1	1						1		
FFP, units (1 unit−120 ml)	1	1		1							1		
Brain NIRS right hemisphere, %	60	64	65	64	78	79	81						
Brain NIRS left hemisphere, %	61	62	82	83	91	90	92						
Transplant NIRS, %	74	81	90	91									
Hb, g/L	72	84	76	63	69	97	100	66	60	89	75	76	
WBC, × 10^9^/L	1.25	9.63	15.48	7.89	9.26	7.55	7.54	9.99	10.45	11.97	21.18	19.32	
Platelets, × 10^9^/L	11	79	29	6	7	12	7	13	10	12	13	13	
ALT, IU/L	20.5	2108.7	914.6	273.5	21	70.9	30.9	20	17.1	15.4	13.2	10	7.9
AST, IU/L	31.9	3172.4	4038.3	1291.5	265.4	647.3	237.9	97.9	60.4	50.6	46	37.2	24.5
Albumin, g/L	28.7	10.2	31.7	14.9	21.4	26.6	35	26	22.3	22.5	19.7	22.6	25.7
Creatinine, mmol/L	340	95	95	86	20	116	101	85	62	73	78	57	4
Urea, mmol/L	31.19	12.72	12.25	11.82	12.26	12.68	11.5	9.52	8.73	12.13	15.22	11.86	10.08
LDH, U/L	327	5,863	3,405	1,540	281	1,229	907	596	128	92	92	447	405
Creatine kinase, U/L		2,410	7,867	10,468	12,425	3,677		300	509	510	522	58	47
C-reactive protein, mg/L	15.77	8.95	109.38	67.81	128.44	137.72	129.88	97.27	1.88	64.65	126.57	128.22	62.82
Procalcitonin, ng/ml	3.12	6.54	9.82	0.58	3.62	3.78	5.47	2.85		2.08	91.8	47.72	19.69
CVVHD filtrate volume, ml/day		500	1,500	1,200	1,230	1,300	1,700	670					

*D0, day of transplantation; HR, heart rate; CVP, central venous pressure; BP, arterial blood pressure; MAP, mean arterial pressure; ECMO, extracorporeal membrane oxygenation; RBC, red blood cells; FFP, fresh frozen plasma; NIRS, near infrared spectroscopy; Hb, hemoglobin; WBC, white blood cells; ALT, alanine aminotransferase; AST, aspartate aminotransferase; LDG, lactate-dehydrogenase; CVVHD, continuous venovenous hemodialysis; PE, plasma exchange*.

Plasma exchange (PE) was started as soon as hemodynamics were stabilized. The purpose was to prevent thrombocytopenia—associated multiorgan failure (TAMOF) and to decrease ALT, AST, LDH, and CK levels. PE was performed according to the institutional protocol: two volumes 16 h after the ECMO start, and one volume 40 h since the ECMO initiation. To keep up the immunosuppression we infused additional 300 mg of eculizumab after each PE session. CRRT in CVVHD mode (Fresenius Medical Care, hemofilter Ultraflux AVpaed) was started 18 h after the ECMO initiation. The CRRT circuit was pre-filled with erythrocytes and crystalloids—this helped to reduce additional eculizumab dosages as plasma transfusions were not needed between extracorporeal detoxication sessions. We used a restrictive transfusion strategy in this clinical case with goal Hb level 70–90 g/L and used transfusion for extracorporeal circuit and the detoxication devices volume compensation only.

The therapeutic effect of PE was insufficient (a slight decrease in ALT, LDH, rise of AST, CK, CRP, PCT, and vasopressor requirement) and blood purification with CytoSorb (Cytosorbents, USA) was initiated on POD 2. The goal was to stabilize hemodynamics, reduce vasopressor requirement and eliminate high concentrations of inflammatory mediators and ECMO-induced free hemoglobin. CytoSorb was installed into the CRRT circuit before the hemofilter. The CRRT access line was connected to the ECMO venous line and the return flow was through the CRRT venous line to the oxygenator bubble trap. The blood flow rate for CytoSorb was 80 ml/min; the procedure duration was 21 h (Table 1): for 12 h on POD2 and 9 h on POD3.

**Figure 1:**
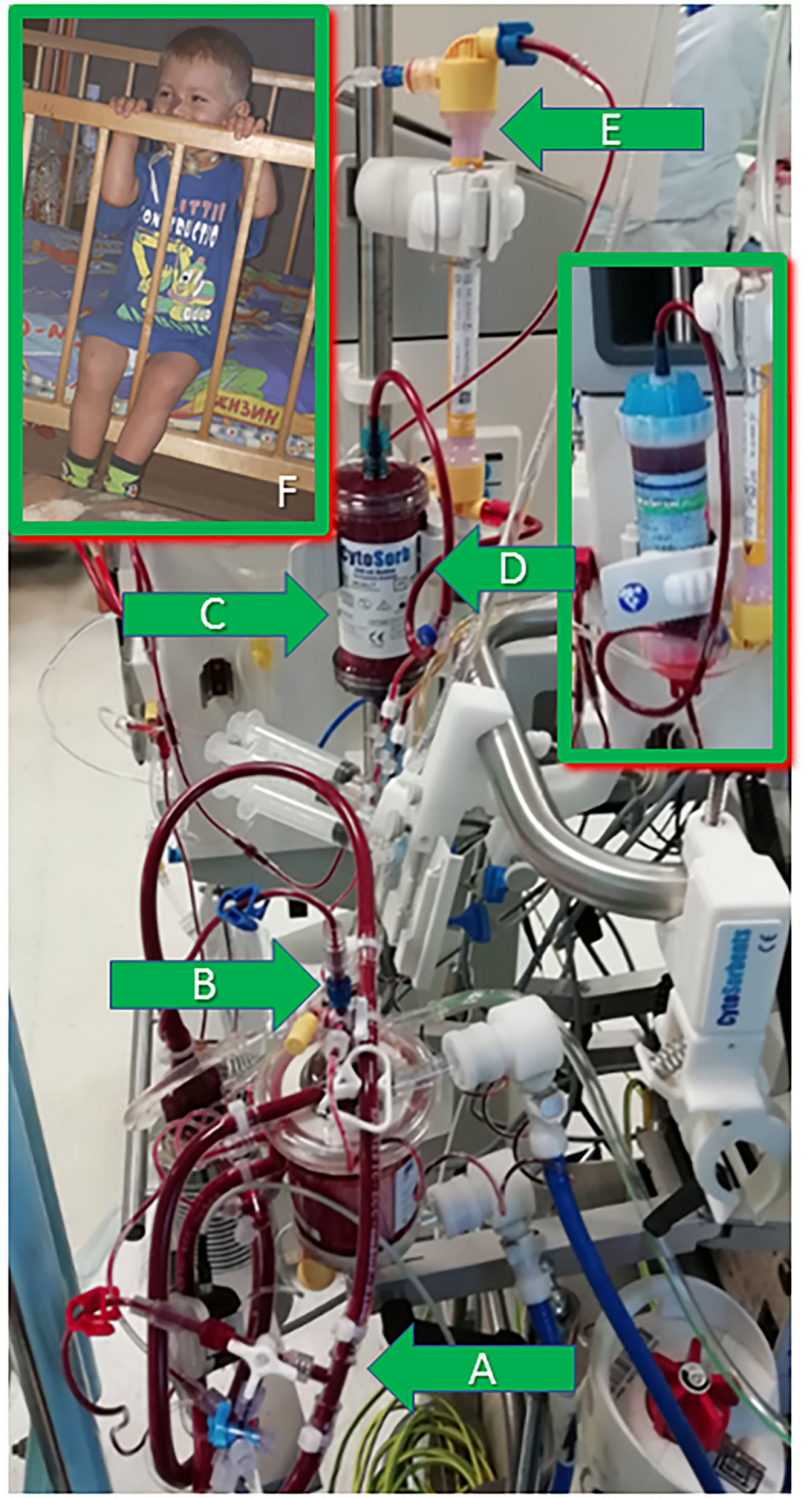
The extracorporeal circuit illustration and the patient 1 year post-surgery. **(A)** The CRRT machine arterial line—to the ECMO system venous line. **(B)** The CRRT machine venous line—to the air trap of the ECMO oxygenator. **(C)** The CytoSorb adsorber installed pre-filter into the CRRT circuit. **(D)** PMX-20R column installed later in the same position—pre-filter in the CRRT circuit. **(E)** Pediatric hemofilter in the CRRT circuit. **(F)** The patient 1 year post-surgery.

While the CytoSorb therapy successfully suppressed inflammation (measured by CRP and PCT decrease), but the increased inotropic requirements suggested uncontrolled infection (later identified as abdominal translocation of polyresistant Klebsiella Pneumoniae, discovered in feces). The suspected infection was treated with antibiotic regimen escalation, Colistin was added to Tienam/Linezolid and three sessions of lipopolysaccharides adsorption with the PMX-20R column (Toray, Japan) to avoid lipopolisaccharides endotoxic action (Figure 1) on POD 3, POD 4 and POD 10 (as PCT level rose again 3 days after the abdomen closure) (8). The PMX-20R column was installed into the circuit pre-hemofilter in the same way as described above. The blood flow rate was 100 ml/min. The procedure duration was 8 h.

The use of these extracorporeal therapies and appropriate antibiotic regime controlled the infection. The hemodynamics stabilized quickly and inotrope index decreased from 165 to 10 within 2 days, ECMO could be discontinued on POD 6, decannulation was uncomplicated. We closed the abdomen on POD 7, diuresis restored on POD 8. CRRT could be discontinued on POD 15, ventilation could be ceased on POD 60.

The total ICU stay was 92 days, the patient was discharged from hospital on POD 116 with a moderate neurological deficit and a stable graft function (creatinine 51 mcmol/l, proteinuria 9 mg/l). In follow-up observation (1 year after surgery) the kidney graft function remained stable all this time: creatinine level 54 mcmol/l, proteinuria 8 mg/days. Despite much secondary damage to the kidney graft it functioned well on standard immunosuppression: tacrolimus 0.5 mg × 3 times/day to reach blood concentrations of 3–4 ng/ml, MMF 125 mg × 2 times daily.

Twelve months after surgery the neurological improvement was evident, though pre-transplant co-morbidities hindered the recovery (osteoporosis prevented walking, tracheostomy complicated speech). The patient could sit, monitor the surroundings, actively used a spoon, entertained toys and examined pictures in children books. He maturely showed emotions, recognized and differentiated his father and mother and related negatively to staff.

## Discussion

Here we describe a case of using ECMO in conjunction with multimodal extracorporeal therapy in a successful treatment of an otherwise hopeless pediatric patient. As the efficacy of extracorporeal detoxication in adults is still undergoing investigation, there are even fewer studies examining its use in pediatric patients. The aim of this report is to demonstrate that such a combination is feasible and can provide a favorable patient outcome.

Despite ECMO, PE, and CRRT it was evident that our patient was going into MODS. Given the multi-organ failure and the nature of the therapy (ECMO, extended venous hemodialysis, immunosuppression, infusion therapy) we had to choose the safest tactics. We chose to apply novel therapies to achieve adequate control of inflammation and to prevent and treat MODS. However, the patient course suggested that there was an ongoing infection, so we used PMX-20R to control it and simultaneously adsorb endotoxin.

CytoSorb has been shown in multiple studies and case reports to effectively eliminate cytokines and DAMPs (9), reduce vasopressor requirements, limit endothelial damage and play a positive role in survival in adults (10, 11). In the observational study of eight pediatric patients Bottari et al. demonstrated that CytoSorb used in conjunction with CRRT was associated with a fast and significant decrease in catecholamine demand, hemodynamic stabilization and an improvement in vasoactive-inotropic score (12). Calabrò et al. demonstrated the efficacy of blood purification with CytoSorb in adult patients on extracorporeal life support (ICU mortality decrease from the expected 80–52.3%) (13). Borzacchi et al. described successful use of CytoSorb in the context of pediatric ECMO, stating that it allowed for a fast healing from anuria and reperfusion damages after refractory cardiac arrest (14). In our case, CytoSorb efficiently reduced inflammatory markers, ALT, AST, LDH, CRP, and PCT levels, but the patient continued to be hemodynamically unstable. We would like to stress the point that the hemodynamic instability after CytoSorb is not the terminal stage for the patient, but rather a clear indication for further search for an unidentified infection.

We suspected gram-negative shock, so we escalated the antibacterial therapy and started lipopolysaccharides adsorption, which is reported to be successful in endotoxin removal (15–17). Hirakawa et al. demonstrated that polymyxin B-immobilized fiber contained in the PMX-20R column successfully adsorbs endotoxin in a pediatric patient (18). Kim et al. described a case of the successful application of polymyxin B-immobilized fiber column hemoperfusion to a neonate on extracorporeal membrane oxygenation (ECMO) support (19). This lipopolysaccharide adsorption technique is capable of binding and neutralizing endotoxin and allows for safe use of the polymyxin antibiotic, which is toxic when used systemically (18, 20). Timely endotoxin removal is crucial when high potency antibiotics are involved in intensive care. This approach has been validated and found successful in the retrospective analysis treatment of adult patient population with septic shock (21).

We used a restrictive transfusion strategy during ECMO in this clinical case and consider this as one of the reasons for the favorable outcome. The evidence in favor of such an approach is extensive (5, 22): two retrospective observational studies have been published recently on the restrictive transfusion strategies (23, 24); in a study by Omar et al. the volume of transfusion was shown to be higher in non-survivors compared to survivors (25). The kidney graft perfusion was started in the setting of the running ECMO. Therefore, CRRT was initiated with the rationale to treat AKI and fluid overload (26). The use of RRT is reported to have a positive influence to reduce ECMO duration (27). Yet, it is well-known that CRRT does not eliminate cytokines, while they play an important part in MODs progression. In our case PE was not sufficiently effective in removing significant quantities of inflammatory mediators. Some of the inflammatory markers' concentrations in fact increased after the PE sessions (Table 1). Thus, starting the adsorption therapy was the safest and the most beneficiary approach: it allowed for clinical stabilization, controlled MODS and supported a positive clinical outcome.

The technical connection of extracorporeal therapies to the ECMO circuit was feasible and did not affect the flow of the ECMO system and did not lead to increased clotting.

The limitation of this study is that this is a description of a single case. However, this is the first case report of successful combined use of extracorporeal technologies in a child with MODS, major immunosuppression and ECMO associated with a good outcome.

## Conclusions

Contemporary methods of extracorporeal life support allow for stabilization in previously hopeless cases. Combined use of adsorption technologies together with ECMO can be safe and feasible in children. An important finding is that hemodynamic instability after CytoSorb is a clear indication for further search of infection source rather than a terminal sign. Studies are needed to evaluate the use of these therapies for the management of MODS after ECMO resuscitation. There should be increased focus on the development of adsorbers with smaller priming volumes for children.

## Data Availability Statement

The original contributions presented in the study are included into the article, further inquiries can be directed to the corresponding author.

## Ethics Statement

Written informed consent for the publication of the data and any potentially identifiable images was duly provided by the minor's legal guardian/next of kin. The ethical review and/or approval was not required for the publication of the data.

## Author Contributions

All authors listed have made a substantial intellectual contribution to the work, commented the manuscript, read and approved the final text, and have approved it for publication. Data collection and discussion were performed by RA, AP, and MK. The data analysis was performed by NR and AB. The manuscript was drawn by AR. The overall manuscript revision was performed by VL.

## Conflict of Interest

The authors declare that the research was conducted in the absence of any commercial or financial relationships that could be construed as a potential conflict of interest.
